# A 2,000–year-old specimen with intraerythrocytic *Bartonella quintana*

**DOI:** 10.1038/s41598-020-66917-7

**Published:** 2020-06-22

**Authors:** R. Barbieri, B.-H.-A. Mai, T. Chenal, M-L. Bassi, D. Gandia, L. Camoin-Jau, H. Lepidi, G. Aboudharam, M. Drancourt

**Affiliations:** 1 IHU Méditerranée Infection, Marseille, France; 2 Aix-Marseille-Université, IRD, MEPHI, IHU Méditerranée Infection, Marseille, France; 3 Aix-Marseille Univ, CNRS, EFS, ADES, Marseille, France; 4 Hue University of Medicine and Pharmacy, Hue, Vietnam; 5 Ville de Besançon DPH, CNRS, UMR 6298 ArTeHiS, Marseille, France; 6 Laboratoire d’Hématologie, Hôpital de la Timone, APHM, Marseille, France; 7 Service d’Anatomopathologie, Assistance Publique des Hôpitaux de Marseille, Marseille, France; 8 Aix-Marseille-Université, UFR Odontology, Marseille, France

**Keywords:** Microbiology, Bacteria, Pathogens

## Abstract

Photogrammetry and cascading microscopy investigations of dental pulp specimens collected from 2,000-year-old individuals buried in a Roman necropolis in Besançon, France, revealed unprecedented preserved tissular and cellular morphology. Photogrammetry yielded 3-D images of the smallest archaeological human remains ever recovered. Optical microscopy examinations after standard haematoxylin-phloxine-saffron staining and anti-glycophorin A immunohistochemistry exposed dental pulp cells, in addition erythrocytes were visualised by electron microscopy, which indicated the ancient dental pulp trapped a blood drop. Fluorescence *in situ* hybridisation applied on red blood cells revealed the louse-borne pathogen *Bartonella quintana*, a finding confirmed by polymerase chain reaction assays. Through paleohistology and paleocytology, we demonstrate that the ancient dental pulp preserved intact blood cells at the time of the individual’s death, offering an unprecedented opportunity to engage in direct and indirect tests to diagnose pathogens in ancient buried individuals.

## Introduction

Teeth are organs that can be recoverable in human individuals from around ~300,000 years ago^1^ and could be the last organs that remain intact in dead individuals^2,3^. Teeth contain dental pulp on which fruitful paleomicrobiology investigations have been performed^4^. Indeed, intact ancient dental pulp preserves ancient pathogen biomolecules such as proteins and DNA, under favourable biochemical conditions such as freezing or rapid desiccation of tissues which partially inhibits DNA degradation (combined with favourable humidity, temperature, pH and salinity^5^) but not hydrolytic and oxidative processes^6^. From the ancient dental pulp, genome-wide data were analysed from various micro-organisms including bacteria, viruses and parasites^7^, such as the plague agent *Yersinia pestis*, the relapsing fever agent *Borrelia recurrentis*, the leprosy agent *Mycobacterium leprae*, the typhoid fever pathogen *Salmonella enterica*, the hepatitis B virus and the malaria agent *Plasmodium falciparum*. In addition, dental pulp preserves ancient proteins in such a way that the paleoproteomics of pathogens can be performed^8^. Recent investigations unexpectedly revealed host peptides, including peptides derived from conjunctive dental pulp tissue and plasmatic peptides, such as coagulation factors and immunoglobulins^8^. These observations indicated that ancient dental pulp can preserve blood and its serum phase.

Here, using paleocytology, we show that ancient dental pulp can also preserve blood cells exhibiting unanticipated and perfectly conserved morphology. Illustrating one outcome of this discovery in the field of paleomicrobiology, we performed microscopic-only detection of one ancient intracellular pathogen, *Bartonella quintana*^9^, in a 2,000-year-old Roman dental pulp specimen obtained in France.

## Results

### Photogrammetry

**Figure 1:**
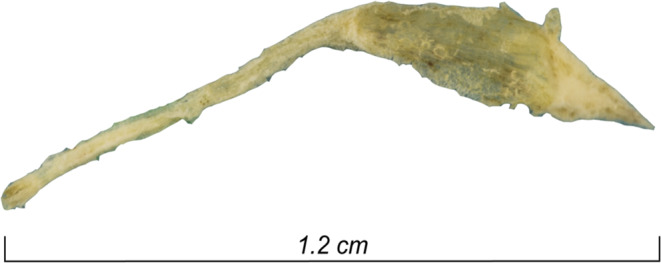
Photogrammetry of a 2,000-year-old intact dental pulp recovered from Ind. 17. qPCR-based detection of *B. quintana*.

A total of 25 teeth were collected from five individuals (here referred to as individuals 17, 20, 21, 33 and 35) discovered in a 2,000-year-old Roman archaeological site (Viotte Nord, Besançon) in France. The dental pulp was recovered from each tooth as previously described, and two dental pulp aliquots were prepared^10,11^. The dental pulp recovered from individual 17, which was macroscopically intact, was further analysed by photogrammetry. A total of 1,500 images enabled us to recreate the pulp sample in full volume at the following photogrammetry specifications: a scattered cloud of 104.256 points, a dense cloud of 361.35 points, a mesh volume of 822.771 faces, and finally a mosaic texturing of 12.288 ×12.288. The quality of each photo thus made it possible to observe the dental pulp, with a rendering that was close to being observable with the naked eye or binocular glasses, as shown in Fig. 1.

A total of 24 dental pulp specimens collected from macroscopically intact teeth presenting with a closed apex and no abrasion, from individuals 20, 21, 33 and 35 were tested for the presence of *B. quintana* DNA (Despite a high prevalence among ancient populations, this pathogens does not leave any characteristic traces on bones^12^) by detecting the *yop*P^13^ and intergenic transcribed spacer (ITS)^14^ sequences by real-time PCR, as previously described^11^. With the negative controls remaining negative, one dental pulp sample collected from individual 35 was positive for two of the molecular targets, 2/24 dental pulp samples were positive for *yop*P only, and 7/24 dental pulp samples were positive for the ITS region only. Therefore, individual 35 was determined to be *B. quintana*-positive; individual 33 was determined to be *B. quintana*-negative (Table 1). The pulp from both individuals was further investigated by microscopy as described.

**Table 1:** Overview of *yopP* and ITS detection in samples from individuals 20, 21 and 35 whose dental pulp specimens positive for *yopP* or/and ITS (+) and negative specimens (–).

Indivudual	N° Tooth	*yopP*	ITS	Cq
21	1	+	–	37.8
	2	–	+	37.4
	3	–	+	38.2
	4	+	–	33.2
	5	–	+	34.2
20	8	–	+	35.3
	9	–	+	34.4
	10	–	+	34.8
35	13	–	+	36.2
	14	+	+	33.4; 34.5

Individual 33 was negative for both PCR targets and is not presented in this Table.

### Paleocytology

**Figure 2:**
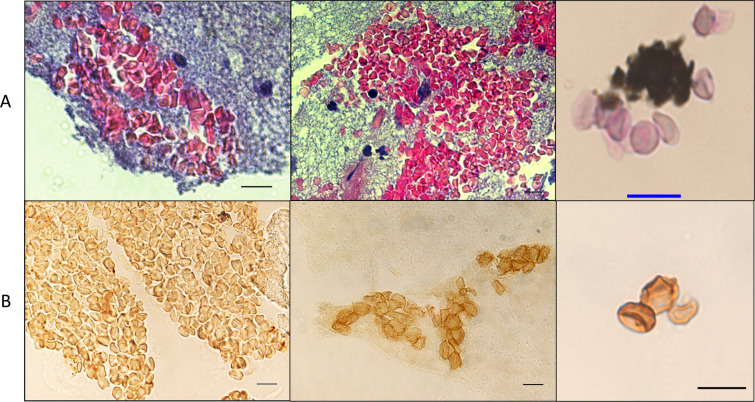
Identification of ancient erythrocytes in individual 33 (*B. quintana*-negative) and individual 35 (*B. quintana*-positive) (**A**) HPS staining (10-µm scale) and (**B**) anti-glycophorin A staining (10-µm scale).

The dental pulp specimen collected from the *B. quintana*-positive individual (35) was immersed in a rehydration buffer, adapted from Sandison *el al*.^15^, consisting of 1% formalin, 96% ethanol and 5% ammonium bicarbonate for 24 hours before haematoxylin-phloxine-saffron (HPS) staining and anti-glycophorin-A staining, which is specific for detecting erythrocytes^16,17^. The microscopic observation was performed from 10× to 100× magnification. In these experiments, the dental pulp sample collected from the *B. quintana*-negative individual (33 was used as the negative control and manipulated strictly in parallel to the one the dental pulp sample collected from the *B. quintana*-positive individual (35). Microscopic observations of the HPS-stained dental pulp specimens (five slides for individual 35 and five slides for individual 33) unexpectedly yielded the presence of pink or red cells with sizes and morphological characteristics consistent with those of erythrocytes (Fig. 2A).

**Figure 3:**
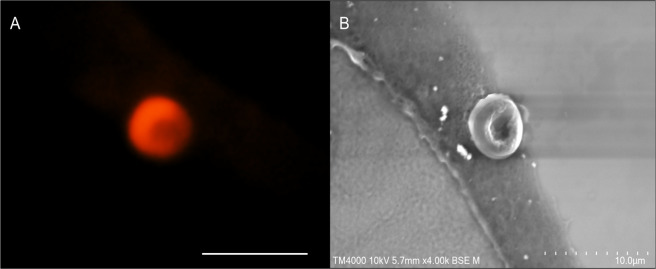
(**A**) Fluorescence microscopy of an erythrocyte in Ind. 35 using wavelength 555 nm. (**B**) The same erythrocyte was observed by scanning electron microscopy (10-µm scale).

Anti-glycophorin A staining (10 slides for individual 35 and 10 slides for individual 33) (Fig. 2B) yielded red-orange-brown coloured cells devoid of nuclei, ranging in size from 2 to 8 µm, confirming these cells as erythrocytes. The stained erythrocytes presented different shapes with no evidence of eryptosis, ranging from round to square (probably due to the cutting edge resulting from the slide preparation), and some cells present concave surfaces specific of erythrocytes, while these morphological characteristics were not observed in the negative controls obtained through the use of an irrelevant antibody. Ancient erythrocytes appeared as separate cells or agglutinated in erythrocytic islets (Fig. 2 and Fig. 3A). Electron microscopy confirmed the remarkable conservation of the erythrocyte morphology 2,000 years after the death of the individual (Fig. 3B).

### Fluorescence *in situ* hybridisation of *B. quintana* in ancient erythrocytes

**Figure 4:**
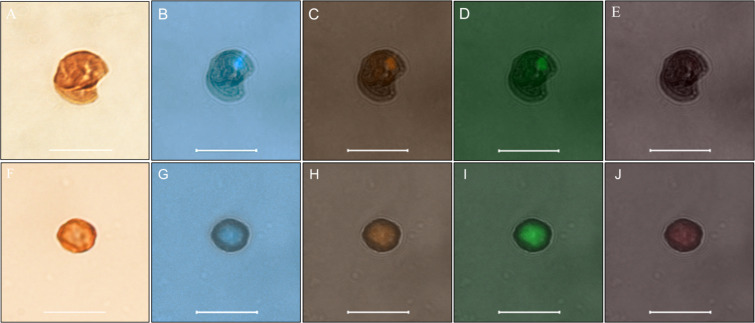
FISH revealed *B. quintana*-infected erythrocytes from individual 35, with a *B. quintana*-negative autofluorescent erythrocyte from individual 33 used as a negative control. (**A–F**): Optical microscopy; (**B–G**): confocal microscopy with DAPI staining. (**C–H**): confocal microscopy with EUB probe. (**D–I**): Confocal microscopy with a *B. quintana*- specific probe. (**E–J**): Confocal microscopy with a nonEUB probe.

The detection of morphologically intact erythrocytes in the dental pulp of two individuals, 33 (negative control) and 35 (*B. quintana* positive), provided a unique opportunity to test for microscopically identifiable intraerythrocytic *B. quintana* organisms using fluorescence *in situ* hybridisation (FISH). Accordingly, the dental pulp samples were stained using 4’,6’-diamidino-2-phenylindole (DAPI), a DNA-binding dye, here used to screen erythrocytes for the presence of exogenous, presumably bacterial, DNA. Indeed, *B. quintana* is acknowledged as such an intraerythrocytic organism^18^, which has been previously detected in ancient dental pulps and bones collected from several archaeological sites in France^19–23^. Erythrocyte autofluorescence (anticipated to interfere with the FISH detection of *B. quintana*) was not observed in erythrocytes, which exhibited a flat morphology (Fig. 4).

**Figure 5:**
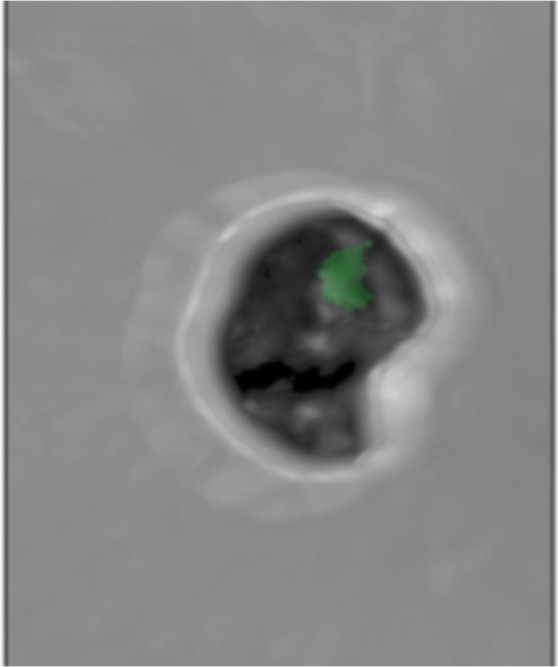
3D-FISH revealed *B. quintana* inside individual 35 erythrocytes under a green channel-specific probe.

Confocal microscopy revealed that one such flat erythrocyte in individual 35 had been stained blue with DAPI, stained red with the pan-bacterial probe and stained green with the *B. quintana*-specific probe, remaining dark with the non-specific probe, whereas no such images were observed in the sample from negative-control individual 33, indicating that bacteria were present in the erythrocytes of individual 35 and that these bacteria were *B. quintana* (Figs. 4 and 5).

## Discussion

Microscopy was the first ever laboratory tool used in the 19^th^ century for the diagnosis of infectious (bacterial) diseases by the direct observation of microbes in clinical samples^24,25^, and we confirm that microscopy is still a diagnostic approach of value when addressing pathogens in ancient specimens. In this study, all microscopic observations were authenticated by negative controls. Additionally, microscopic detection of *B. quintana* was confirmed by two different qPCR assays, including negative controls and no positive controls.

In this study, the intact preservation of dental pulp blood cells is consistent with few previous observations. Indeed, although the authors had no previous observation of an exceptionally well-preserved dental pulp specimen as reported here despite handling about 5,000 ancient dental pulp specimens spanning 70 centuries over 25 years, nevertheless ancient morphically intact human erythrocytes have been detected from mummified soft tissues up to 5,300 years old, in one 1,600-year-old bone marrow sample^26^ and in Mediaeval bone remains^27^. Erythrocytes of unknown origin have also been microscopically detected on prehistoric archaeological tools potentially used for hunting and meat preparation^28,29^. The fact that mammal erythrocytes can be preserved for thousands of years over other cell lines may be due to the specific structure of the membrane which is enriched in collagen (still largely present around 5,000 years after the death of the individual^30^) possibly enabling elasticity and great resistance. Furthermore, Raman spectroscopy performed on ancient red blood cells suggested associated fibrin peptides and the formation of blood clots^30^.

The paleocytological observations reported here contribute one more specimen (dental pulp) upon which to base the microscopic detection of mammal cells, in this case human erythrocytes, further illustrating that archaeological specimens could preserve morphologically intact blood cells, in addition to serum proteins^31^. Here, we show that ancient teeth entrapped a blood clot following the death of the individual. Combining these observations suggested that indirect (serological) and direct diagnosis tests using blood could be assayed on ancient dental pulps.

As an illustration, paleocytology unexpectedly enabled the microscopic detection of a 2,000 year-old intraerythrocytic *B. quintana* in the same way that has been previously reported in modern blood smears^18^.

The paleocitology approach developed in this study further offers the possibility of experimental testing on infected erythrocyte samples dating from thousands of years ago, the evolutionary history of *B. quintana* only known through the analysis of modern genomes^32^. Indeed, it has been shown through comparative analysis that *B. quintana* is a reductive genome evolution *of B. henselae*, occurring in particular by losing the genomic specific island coding for filamentous hemagglutinin, thus becoming a successful single-vector bacterium, unlike *B. henselae*. In this study, paleocytology makes it possible to access erythrocytes infected with *B. quintana* more than 2,000 years ago, which could contribute to clarifying the timelines of *B. quintana* specialisation through single cell genome sequencing. Our study therefore adds a new approach to the detection of this pathogen that, so far, has only been detected through PCR-based techniques (Table 1). The efficiency of PCR-based approaches could be limited by a risk of laboratory cross-contamination leading to false positive results and the presence of PCR inhibitors leading to false negative results^33^. Microscopic techniques, including FISH, are less prone to such limitations, offering a complementary approach to PCR-based techniques. The observations reported here pave the way to sorting *B. quintana*-infected red blood cells for further characterisation of ancient pathogens, including whole genome sequencing.

This report introduces paleocytology as an innovative approach for the analysis of ancient dental pulp to study both host- and host-associated pathogens, complementing the study of ancient biomolecules^4,7,8,34^.

## Materials and Methods

### The archaeological site

The preventive excavation “Viotte Nord” was carried out as part of work related to a railway station in Besançon in north-eastern France. The archaeological intervention took place over a surface area of 2,786 m2 on which forty burials, six secondary cremation burials and a probable dump area were exhumed. The furniture from funeral structures, as well as C^14^ dating on bones and charcoal in this burial site was attributed to the transition period between the High Empire and the Lower Empire, i.e., I-IV centuries AD (Supplementary Fig. 1). The excavation of funerary materials revealed 43 more-or-less well-preserved skeletons. Most individuals were wrapped in textiles in a wooden container; some individuals were accompanied by grave goods, including ceramics, glass, shoes and animal bones. We received teeth from four individuals collected from three burials (SEP): SEP 230 (Supplementary Fig. 2) was a double-burial site containing one mature man (Ind. 21) and one woman (Ind. 20), with archaeological studies and C^14^ dating, this structure was dated between the 2^nd^ and 3^rd^ centuries; SEP 283 (Supplementary Fig. 3) contained an immature individual (Ind. 33), with archaeological studies dating the structure between the middle 2^nd^ century and the 4^th^ century; SEP 286 (Supplementary Fig. 4) contained a young adult (Ind. 35) of undetermined sex, archaeological studies dating the structure to the 2^nd^ century; SEP 225 (Supplementary Fig. 5) contained a mature adult man (Ind. 17) of approximately 1.74-m stature, C14 analysis dating this individual from between the second and third centuries. We analysed a total of 25 teeth, including seven teeth collected from Ind. 21, five teeth collected from Ind. 20, eight teeth collected from Ind. 35, four teeth collected from Ind. 33 and one tooth collected from Ind. 17.

### Pulp collection and photogrammetry

Teeth collected from five individuals at the site of Viottes were processed in a dedicated laboratory room at the Institute of IHU Méditerranée Infection, Marseille, France. The teeth were brushed under tap water to remove dirt and debris and were finally washed with nuclease-free water. Under a chemical hood, an electric motor with a rotating diamond disc was used to open each tooth longitudinally, dividing it into two parts. Pulp was extracted using a sterile dental excavator and scraped into two sterile Eppendorf tubes: one was maintained for use DNA extraction as previously described^10,11^ and the other was used to prepare microscopic slides as described below. The dental pulp from individual 17 was preserved in such good condition that it was analysed by photogrammetry. To digitise the image as close as possible to the true ancient dental pulp, which was reputed to be transparent, we used precision photogrammetry equipment (Agisoft Photoscan 1.5, Agisoft LLC, Saint Petersburg, Russia) using a 100-mm macro lens and a full frame sensor (EOS 6D Mark II, Canon, Tokyo, Japan). Because of the transparency and smallness of the object, a white light room was installed in the laboratory to obtain controlled light diffusion, as well as to capture images in three focused sectors. A single in-focus snapshot of the entire object using a bracketing technique was generated from a combination of three snapshots developed based on three separate parts of the object.

### DNA extraction and amplification

DNA was extracted with a modified phenol-chloroform protocol^35^. Briefly, each Eppendorf tube containing 5–15 mg dental pulp powder was added to a solution of 10 µL of 25 mg/mL proteinase K, 10 µL of 10% dodecyl sulfate sodium (SDS), and 200 µL of sterile water and agitated at 56 °C overnight before the addition of 220 µL of stabilised phenol/chloroform/isoamyl-alcohol (25:24:1) (Biosolve, Valkenswaard, Netherlands). The tube was centrifuged at 16,000 g for five minutes, and 220 µL of phenol/chloroform/isoamyl-alcohol was mixed with the upper phase for a second extraction. The upper phase was incubated at −20 °C overnight with a solution of 1 µL of glycogen, 44 µL of ammonium acetate (10 M) and 440 µL of absolute ethanol. After a 30-minute centrifugation at 20,000 g at room temperature, the pellet was washed with 440 µL of 70% ethanol, dried at 50 °C for 10 minutes and finally resuspended in 50 µL of nuclease-free water. The DNA extracts were tested for *B. quintana* DNA using quantitative real-time PCR (qPCR) targeting the ITS region and *yop*P genes sequences, as previously described^36^. A dental pulp sample was considered positive when the qPCR reaction was positive with a cycle number (Ct) lower than 40 for at least one gene. The qPCR results guided the choice of one *B. quintana*-positive dental pulp and one *B. quintana*-negative dental pulp sample for subsequent analyses (Table 1).

### Rehydration and slide preparation

The rehydration process was adapted from the protocol originally reported by Sandison [Sandison 1955]. Briefly, 200 μL of a solution containing the dental pulp and 2 volumes 5% ammonium bicarbonate, 5 volumes of 1% formaldehyde and 3 volumes of 96% ethanol was maintained for 24 hours at room temperature. Then, the supernatant was removed following centrifugation at 16.000 g for 5 minutes on a bench-top Eppendorf centrifuge. Dental pulp pellets were collected, fixed in 10% buffered formalin, dehydrated in grade alcohol and embedded in paraffin. Serial 3.5-µm sections were cut from the block and mounted on poly-L-lysine-coated glass slides.

### Anti-glycophorin A and HPS staining

To detect erythrocytes, the paraffin sections were incubated with anti-glycophorin A antibody JC 159 (Mouse Monoclonal Antibody, ref: Mob 066-05, Diagnostic BioSystems, Nanterre, France) at a 1/500 dilution using a Ventana Benchmark autostainer (Ventana Medical Systems, Inc., Tucson, AZ). Briefly, after removing the paraffin and washing them with reaction buffer, the sections were incubated with a primary antibody for 32 minutes at room temperature and then incubated with the reagent from an iVIEW DAB detection kit (Roche Diagnostics, Meylan, France). The sections were counterstained with hematoxylin and post-counterstained with bluing reagent. A negative control incorporating an irrelevant monoclonal antibody was run in parallel. The other paraffin sections were stained with HPS on a Tissue-Tek Prisma autostainer (Sakura, CA, US) and microscopically examined (DM2500 microscope Leica, Vienna, Austria).

### Fluorescence microscopy and transmission electron microscopy

Paraffin was removed from the slices by incubating them for 15 minutes at 65 °C followed by 10 minutes at room temperature in a xylene substitute solution (SafeSolv, Fontenay-sous-Bois, France). The tissue section was emerged in a descending ethanol series (100%, 90%, and 70%; 5 minutes each), rinsed in sterile water and air-dried. The slides were covered with cover slips, sealed with flexible mounting adhesive (Fixogum, Marabu, Germany) and examined with a confocal microscope (LSM800 microscope, Zeiss, Marly-le-Roi, France). After the coverslips were removed, the tissues were observed by scanning microscopy (TM4000 Plus microscope, Hitachi, Tokyo, Japan).

### Fluorescence *in situ* hybridisation (FISH)

We used four probes as previously described:^37^ a 16 S rRNA gene sequence-based pvrobe (5’-AATCTTTCTCCCAGAGGG) specific for *B. quintana*, which was labelled with Alexa-488 (Eurogentec, Angers, France); an Alexa-555-labeled probe (Eurogentec); a universal bacterial 16 S rRNA gene sequence-based probe EUB338 (5′-GCTGCCTCCCGTAGGAGT), used as a positive control; and an Alexa-647-labeled (Eurogentec) nonspecific probe non-EUB (5′-ACTCCTACGGGAGGCAGC), used as a negative control. The same slides used for anti-glycophorin A immunohistochemistry were used to perform FISH. The slides were covered with 10 μL of a solution containing 1 μL of the 16 S probe (10 μmol/L), 1 μL of the EUB-338 probe (10 μmol/L), 1 μL of the non-EUB probe (10 μmol/L) and 1 μL of a solution containing 0.1% Tween 20, 5 μL of hybridization buffer (0.9 M NaCl; 20 mM Tris-HCL, pH 8.0; 30% formamide; and 0.01% SDS) and 1 μL of distilled water. The slides were covered with a coverslip, sealed with adhesive and incubated for 10 minutes at 65 °C and then overnight at 37 °C on a FISH-hybridizer (Dako; Agilent Technologies, Santa Clara, CA). The slides were then immersed in a series of baths with SSC buffer at different concentrations of 4×, 2×, 1×, and 0.5×, for 5 minutes in each bath, and finally, rapidly immersed in a distilled water bath at room temperature. The air-dried slides were stained with DAPI (ProLong Diamond antifade - Fisher Scientific) and examined with a Zeiss LSM800 confocal microscope. The fluorescence of the *B. quintana*-specific probe was read with a green filter, the universal probe EUB338 with an orange filter, the non-EUB probe with a red filter and DAPI with a blue filter. Bright-field images were obtained with an ESID detector excited with an FITC 488-nm laser, images in white and black were generated by using combinations of colour channels. Single images were generated from the acquired 4-µm-thick Z-stack images.

## Supplementary information

Supplementary information.
